# The Incidence and Its Associated Factors Relevant to Brain Radionecrosis That Requires Intervention Following Single or Fractionated Stereotactic Radiosurgery Using Vero4DRT for Brain Metastases

**DOI:** 10.7759/cureus.25888

**Published:** 2022-06-13

**Authors:** Takehiro Yamada, Kazuhiro Ohtakara, Takeshi Kamomae, Junji Itoh, Hideki Shimada, Shunichi Ishihara, Shinji Naganawa

**Affiliations:** 1 Department of Radiology, Toyohashi Municipal Hospital, Toyohashi, JPN; 2 Department of Radiation Oncology, Kainan Hospital Aichi Prefectural Welfare Federation of Agricultural Cooperatives, Yatomi, JPN; 3 Department of Radiology, Nagoya University Graduate School of Medicine, Nagoya, JPN; 4 Department of Radiological Technology, Toyohashi Municipal Hospital, Toyohashi, JPN

**Keywords:** oncology, vero4drt, brain radionecrosis, brain metastases, stereotactic radiosurgery

## Abstract

Purpose: Several factors, including the surrounding brain volume receiving specific doses, have hitherto been reported to correlate with brain radionecrosis (BR) after single or fractionated stereotactic radiosurgery (sSRS or fSRS) for brain metastases (BMs); however, those, especially for fSRS, have not yet been fully elucidated. Furthermore, the clinical outcome data of patients with BM treated with SRS using Vero4DRT are extremely limited. Therefore, this study aimed to demonstrate the incidence of BR requiring intervention (BRRI) and its highly correlated factors.

Materials and Methods: Patients with BMs treated with sSRS or fSRS using Vero4DRT at Toyohashi Municipal Hospital between July 2017 and June 2021 were retrospectively reviewed, of whom patients were available for at least 20 weeks of magnetic resonance imaging follow-up from SRS were included, and analyzed. The prescribed dose fractionation schemes to the planning target volume (PTV) boundary included 24 Gy (sSRS), 35 Gy (5 fractions [fr]), 42 Gy (10 fr), and 30 Gy (3 fr), according to the tumor volume and location. The volume of the surrounding normal brain receiving 84 Gy (V84 Gy, biologically effective dose [BED_2_] based on a linear-quadratic model with an alpha/beta ratio of 2, single-dose equivalent [SDE] to 12 Gy), V112 Gy (BED_2_, SDE to 14 Gy) for all lesions, and all irradiated volume, including gross tumor volume (GTV) receiving 81.6 Gy (81.6 Gy vol., BED_2_) for fSRS were calculated, for which cerebrospinal fluid and bone volumes were cautiously excluded. The diagnosis of tumor progression or BR dominance was based on serial T1/T2 matching.

Results: Sixty patients with 120 lesions (65 treated with sSRS and 55 treated with fSRS) were included in the final analysis, with a median follow-up period of 65 weeks. The local control rate at one year was 87.5%. The cumulative incidence of BRRI within two years was 11.5%. The risk of symptomatic BR was significantly higher for V84 Gy >10 cc (*p *<0.001) and V112 Gy >5 cc (*p *= 0.021). In the fSRS group, the cumulative incidence of Grade 3 BR and those requiring resection was significantly higher for 81.6 Gy vol. >14 cc (*p *= 0.003 and *p *= 0.004, respectively). The coexistence of viable tumor tissue and BR could not be ruled out for enlarging lesions after the nadir response, especially for fSRS, due to a lower BED_10_ to GTV margin (<80 Gy, BED_10_).

Conclusions: Stereotactic irradiation with Vero4DRT provided efficacy and safety comparable to previous linear accelerator series, and most of the dose-volume thresholds for BRRI presented in this study were notably lower than those reported in previous studies. This study suggests that the indication of single and up to 5 frSRS should be limited to far smaller tumors than previously acknowledged to ensure long-term safety and efficacy.

## Introduction

With the advent of pharmacotherapy that penetrates brain metastases (BMs), stereotactic irradiation (STI), including single or fractionated stereotactic radiosurgery (sSRS or fSRS), has gained an ever-increasing role in the management of BMs [1], while prescription dose-fractionation schemes, dose distribution, and irradiation methods have substantially varied between institutions [2-4].

Given the ablative nature of STI, radiographical changes, such as increased enhancement and edema of the surrounding brain, also described as radiation effects, are not infrequent phenomena following STI, although further efforts are required to reduce symptomatic brain radionecrosis (BR), which declines patients’ neurocognitive function. Several factors contributing to BR after STI have been reported, such as tumor volume and location and surrounding brain volumes receiving specific doses (i.e., V12 Gy [cc] for sSRS) [5-8], while those for fSRS have not yet been thoroughly elucidated. Furthermore, symptomatic BR can develop even if the previously suggested dose-volume thresholds are followed.

Vero4DRT (Mitsubishi Heavy Industries Ltd., Tokyo, Japan), a dedicated platform for motion tracking STI for extracranial targets, has been used for STI of BM since 2017 at our hospital. However, data on clinical implementation and outcomes related to intracranial STI using Vero4DRT have been extremely limited. In particular, to the best of our knowledge, there are no clinical outcome data on STI for BM using Vero4DRT.

Therefore, this study aimed to demonstrate the incidence of BR requiring intervention (BRRI) after sSRS and fSRS with Vero4DRT for BMs and gain more insight into the relevant factors susceptible to BRRI.

Part of this study was presented at the 13^th^ annual meeting of the Japan Radiosurgery Society held on February 5, 2022.

## Materials and methods

Patients clinically diagnosed with BMs who were treated with sSRS or fSRS using Vero4DRT at Toyohashi Municipal Hospital between July 2017 and June 2021 were included and retrospectively analyzed, and those who had at least 20 weeks of magnetic resonance imaging (MRI) follow-up from STI were exclusively chosen for this study, as short-term follow-up cases could potentially develop tumor progression and/or symptomatic BR afterward. This retrospective, observational, single-center study was approved by the Ethics Committee of the Toyohashi Municipal Hospital. Participants were assured that they could opt out by phone or by visiting our hospital website.

The indication criteria of STI were patients with less than 10 BMs, the maximum diameter of tumor <5cm, and medical condition tolerable to long-term head immobilization. Post-operative STI of the resection cavity, sequential STI following whole-brain radiation therapy (WBRT), and re-irradiation to the recurrent site after STI were excluded. In principle, we have limited the sSRS indication to ≤15 mm in tumor maximum diameter and excluded critical locations such as the paramotor or brainstem to ensure better local control with less toxicity. We intended to retain at least 24 Gy for a marginal dose of sSRS, which is expected for a one-year tumor control probability of 95% [2]. Furthermore, V12 Gy >5cc, or even 12 Gy volume (all the irradiated volume including tumor) in sSRS is associated with an increased risk of symptomatic BR [9-11], which suggests that suitable indication of sSRS would be far smaller than 2 cm in terms of long-term safety. The dynamic conformal arc (DCA) technique, including noncoplanar arcs, was mainly used for SRS planning with 6 MV X-rays, while step and shoot intensity-modulated radiotherapy (IMRT) was adopted for a few cases with proximity to an organ at risk. The linear accelerator (LINAC) of Vero4DRT is mounted on an O-ring gantry with a restricted angle of a noncoplanar arc or beam that can be delivered without couch rotation [12]. The multileaf collimator consisted of 30 leaf pairs with a 5.0 mm central leaf width. The treatment planning system was the iPlan RT Dose (BrainLAB Feldkirchen, Germany) version 4.5.5, and the dose calculation algorithm was a pencil beam with radiological path-length correction. Each positional verification and correction setup was performed using the ExacTrac system (BrainLAB, Munich, Bavaria) and a robotic couch capable of six degrees of freedom correction. Contrast-enhanced computed tomography images were co-registered with gadolinium-enhanced T1-weighted magnetic resonance imaging (Gd-T1 MRI). Gross tumor volume (GTV) was defined as an enhancement lesion on Gd-T1 MRI or a visible mass lesion on T2-weighted images with weak enhancement or prominent exudation into the surrounding brain. The planning target volume (PTV) was generated by a 1-2 mm symmetrical margin to the GTV (1 mm for sSRS, 1-2 mm for fSRS). The prescription dose at the PTV boundary covered by the 80% isodose surface, normalized to 100% at the isocenter, was 24 Gy for sSRS, while those for fSRS included 35 Gy for 5-fraction (fr), 42 Gy for 10 fr, and 30 Gy for 3 fr. The PTV coverage value is usually ≥98%. To improve dose conformity, a modified PTV structure was frequently used as a surrogate object for MLC fitting [13]. The biologically effective dose (BED) based on the linear-quadratic formula with alpha/beta ratios of 10 and 2 for the antitumor effect and late normal tissue effect (BED10 and BED2, respectively) was adopted to compare different dose fractionation schemes. GTV D98, a dose covering at least 98% of the GTV, was measured as BED10 according to suggestions [14,15].

The location grade was defined for each target as previously proposed as follows: superficial, involving the area at a depth ≤5 mm from the brain surface; deep, located at a depth >5 mm from the brain surface; central, located in the brainstem, cerebellar peduncle, diencephalon, or basal ganglion [8]. The surrounding brain parenchyma for each lesion was accurately contoured by excluding the cerebrospinal fluid cavity and cranial bone to ensure a precise measurement of the volume that receives specific doses.

The surrounding normal brain volumes (excluding the GTV) that received 84 Gy (BED2, V84 Gy, and single-dose equivalent [SDE] to 12 Gy) and 112 Gy (BED2, V112 Gy, and SDE to 14 Gy) were measured for all lesions.

Additionally, the entire irradiated volume, including the GTV receiving 84 Gy (BED2), referred to here as the 84 Gy volume (vol.), was also computed for sSRS, while 81.6 Gy (BED2) vol. (81.6 Gy vol., equivalent to 24 Gy in 5 fr) was computed for fSRS. The 24 Gy vol. in 5 fr SRS >20 cc correlates with an increased risk of BR, as recently suggested [11].

In principle, patients were examined clinically and imaged radiographically using Gd-T1 and T2 MRI at one and three months after STI and then every 3-4 months. The imaging and clinical follow-up times were defined as the period from STI to the final MRI evaluation date and the last visit or death confirmation date, respectively. In cases with enlarging lesions following the nadir response, the diagnosis of the dominance of tumor progression or BR was based on chronological changes in the combination of Gd-T1 and T2 images, proposed as T1/T2 matching [16] and positron emission tomography for some difficult cases.

The severity of BR was graded according to the Common Terminology Criteria for Adverse Events version 5.0: Grade 1 was asymptomatic; Grade 2 was symptomatic and required steroid therapy; Grade 3 was a severe symptomatic or required medical intervention, and we defined the need for surgical debulking or administration of bevacizumab as refractory to steroids.

The local control (LC) of each treated lesion was defined as free of tumor progression, determined based on the recommendation of the Response Assessment in Neuro-Oncology (RANO) group in 2015 [17], which defines tumor progression as an increase in lesions of ≥20% with an absolute value of ≥5 mm in maximum diameter from the nadir response.

Overall survival (OS), LC, and cumulative incidence of BR from the commencement of SRS were estimated using the Kaplan-Meier method, and the log-rank test was used to compare the subgroups, for which the LC and BR events were scored per lesion. Demise before each event was considered a competing risk. The collection of follow-up data was completed in December 2021.

The correlation of BRRI with GTV diameter and size, location, V84 Gy, V112 Gy, and 84 Gy vol. and 81.6 Gy vol., a combination of chemotherapy or immunotherapy was examined using univariate analysis with Gray’s test and multivariate analysis with Fine-Gray proportional hazards regression analysis.

All p-values were calculated using two-tailed tests, and the criterion for statistical significance was set at p <0.05. All statistical analyses were performed using EZR software (Saitama Medical Center, Jichi Medical University, Saitama, Japan) and a graphical user interface for R (The R Foundation for Statistical Computing, Vienna, Austria).

## Results

Among the 112 cases with 234 tumors, 60 patients with 120 lesions were included in the final analysis. Most of the excluded cases could not be fully evaluated radiographically due to deteriorated medical conditions or immediate transfer to hospice care following STI, although none of the excluded cases available for post-STI images developed overt BR or tumor recurrence in less than 20 weeks.

Descriptive statistics of the demographic and clinical characteristics of the patients are summarized in Table 1.

**Table 1:** Characteristics of 60 patients KPS: Karnofsky performance status; LCNEC: Large cell neuroendocrine carcinoma; RPA: recursive partitioning analysis; STI: stereotactic irradiation. Values are presented as median (range) or number (%).

Characteristics	Value
Age (years)	71 (31-87)
Sex	
Male	34 (56.7)
Female	26 (43.3)
KPS (%)	
100	6 (10.0)
90	23 (38.3)
80	23 (38.3)
70	5 (8.3)
60	3 (5.0)
RPA class	
1	4 (6.7)
2	53 (88.3)
3	3 (5.0)
Whole-brain radiation therapy	
Before STI	4 (6.7)
After STI	6 (10.0)
Primary tumor	
Lung cancer	45 (75.0)
Breast cancer	5 (8.3)
Rectal cancer	3 (5.0)
Endometrial cancer	2 (3.3)
Esophageal cancer	1 (1.7)
Kidney cancer	1 (1.7)
Sigmoid colon cancer	1 (1.7)
Cervical cancer	1 (1.7)
Testicular cancer	1 (1.7)
Histology of primary tumor	
Adenocarcinoma	47 (78.3)
Squamous cell carcinoma	5 (8.3)
Small cell carcinoma	3 (5.0)
LCNEC	1 (1.7)
Clear cell carcinoma	1 (1.7)
Other	3 (5.0)
Imaging follow-up in all patients (week)	58 (20-222)
Clinical follow-up in living patients (week)	72 (25-227)

The preponderance of the primary site and its pathological diagnosis was lung adenocarcinoma.

**Figure 1:**
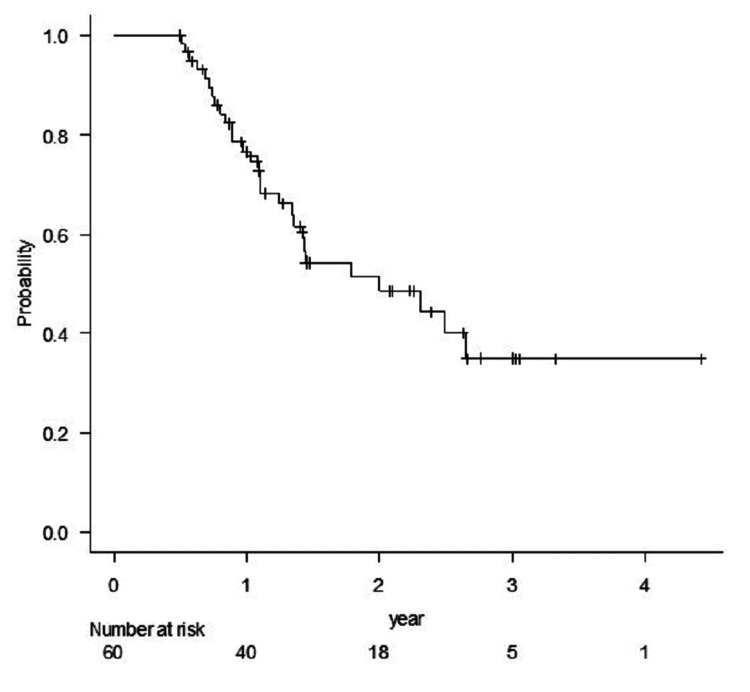
Kaplan-Meier estimate of overall survival of all patients

Whole-brain radiation therapy (WBRT) was administered before STI in 6.7% (four patients with eight lesions) and after STI in 10% of patients (six patients with 11 lesions). The median imaging follow-up time for all patients was 58 weeks (range, 20-222 weeks), and the median clinical follow-up time for survivors (32 patients) was 72 weeks (range, 25-227 weeks). For reference, the 1-and 2-year OS rates were 76.6% and 48.6%, respectively (Figure 1).

The descriptive statistics of the target volumes and dosimetric parameters are presented in Table 2.

**Table 2:** Characteristics of 120 brain metastases and treatment parameters Values are presented as median (range) or number (%). The p-value shows the results of the comparison using the t-test for continuous data or Fisher’s exact test for categorical data.

Characteristic	All	sSRS	fSRS	p-value
sSRS or fSRS		65	55	
GTV diameter (mm)	10 (3-43)	7 (3-15)	17 (4-43)	<0.001
GTV volume (cc)	0.48 (0.01-21.5)	0.19 (0.01-1.75)	1.84 (0.04-21.5)	<0.001
Prescription dose				
24 Gy/1 fr	65 (54.2)	65 (100)		
30 Gy/3 fr	2 (1.7)		2 (3.6)	
35 Gy/5 fr	49 (40.8)		49 (89.1)	
42 Gy/10 fr	4 (3.3)		4 (7.3)	
GTV D_98_ BED10 (Gy)	99.9 (63.9-111.7)	103.8 (96.1-111.7)	72.8 (63.9-89.2)	<0.001
V84 Gy (cc)	2.75 (0.37-28.71)	1.57 (0.37-5.88)	5.42 (0.66-28.17)	<0.001
V112 Gy (cc)	2.01 (0.29-17.96)	1.22 (0.29-4.33)	3.66 (0.46-17.96)	<0.001
84 Gy vol. (cc)		1.76 (0.4-7.23)		
81.6Gy vol. (cc)			7.29 (0.74-43.35)	
Location				0.043
Superficial	60 (50.0)	33 (50.8)	27 (49.1)	
Deep	52 (43.3)	31 (47.7)	21 (38.2)	
Central	8 (6.7)	1 (1.5)	7 (12.7)	

**Figure 2:**
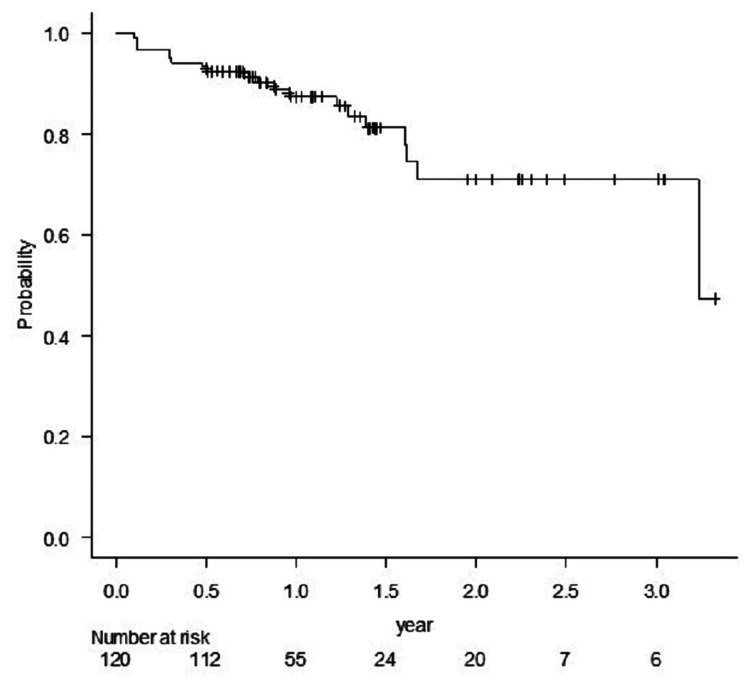
Kaplan Meier estimate of local control of all lesions

sSRS and fSRS were applied to 65 and 55 lesions, respectively. GTV D98 for sSRS was significantly higher than for fSRS (p <0.001). The LC rates for all 120 lesions at one year and one-and-half years were 87.5% and 81.1%, respectively (Figure 2).

**Figure 3:**
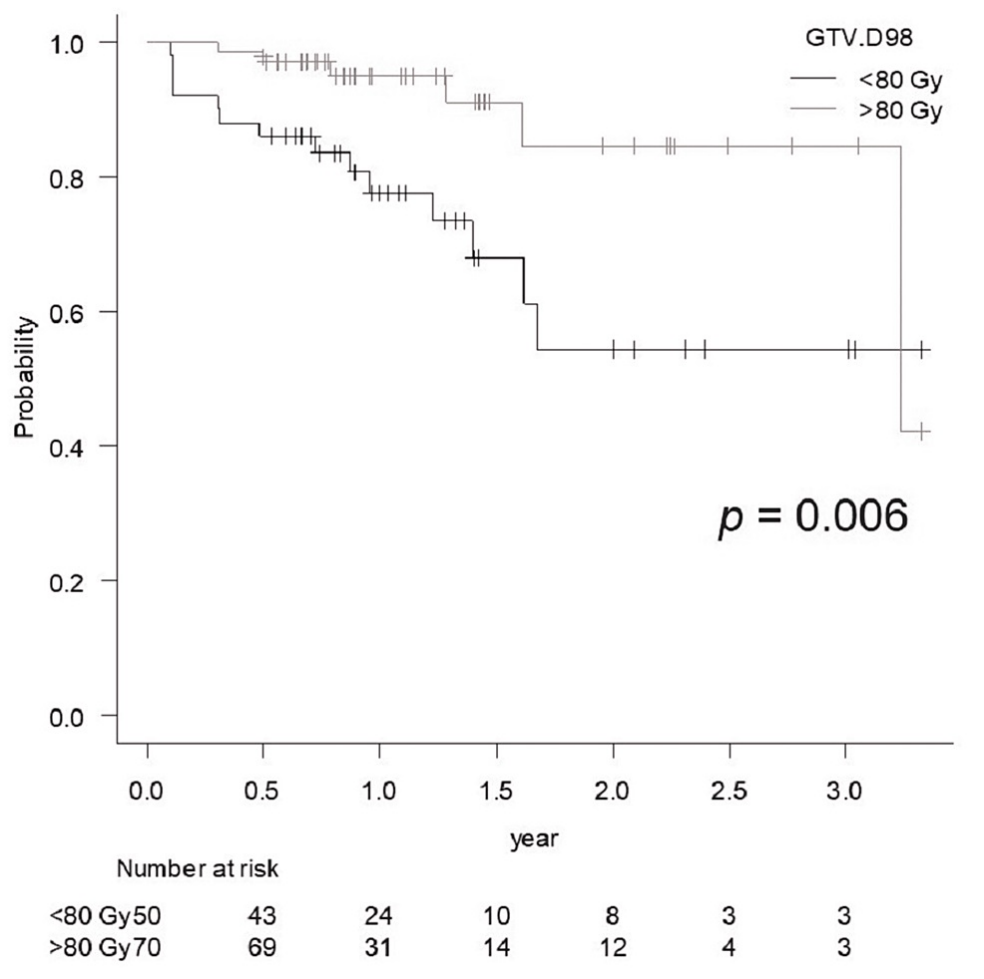
Comparison of the local control rate of lesions treated with GTV D98 <80 Gy vs. >80 Gy

The LC rate for GTV D98 >80 Gy was significantly better than that for <80 Gy (p = 0.006) (Figure 3).

**Figure 4:**
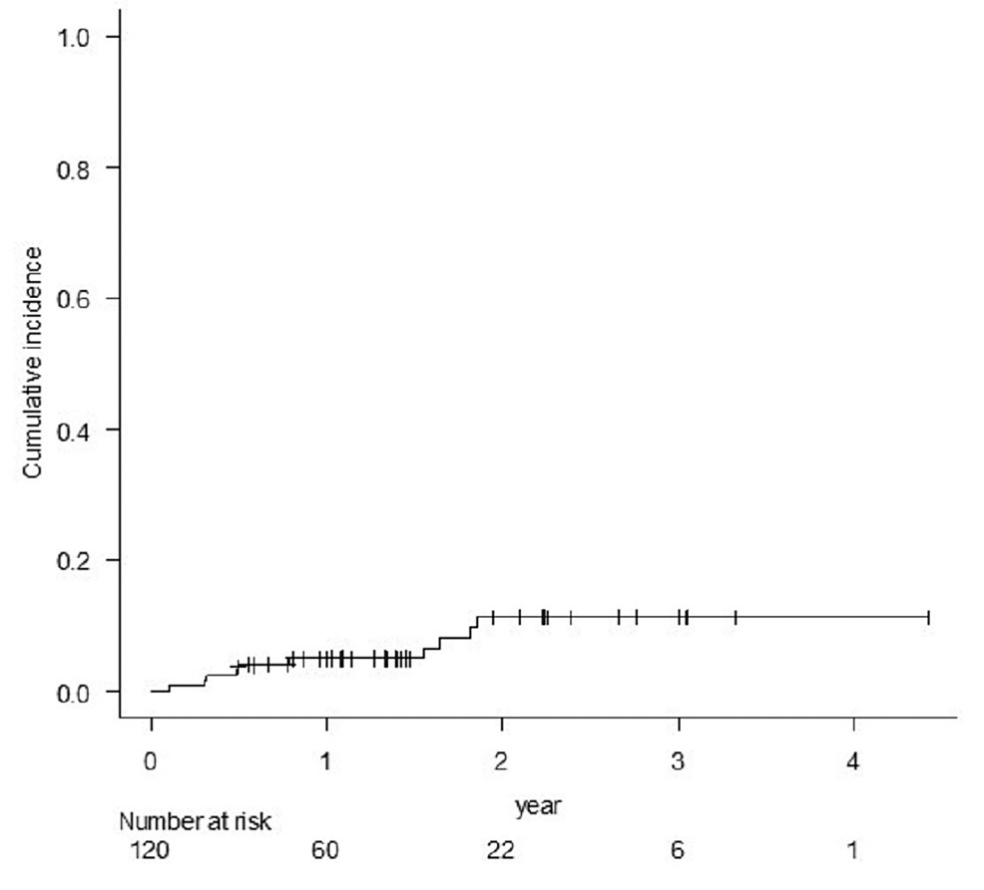
The cumulative incidence rate of brain radionecrosis for Grade 2 or more

The cumulative incidence of BR for Grade ≥2 was 11.5%, and all occurred within 2 years of STI (Figure 4).

**Figure 5:**
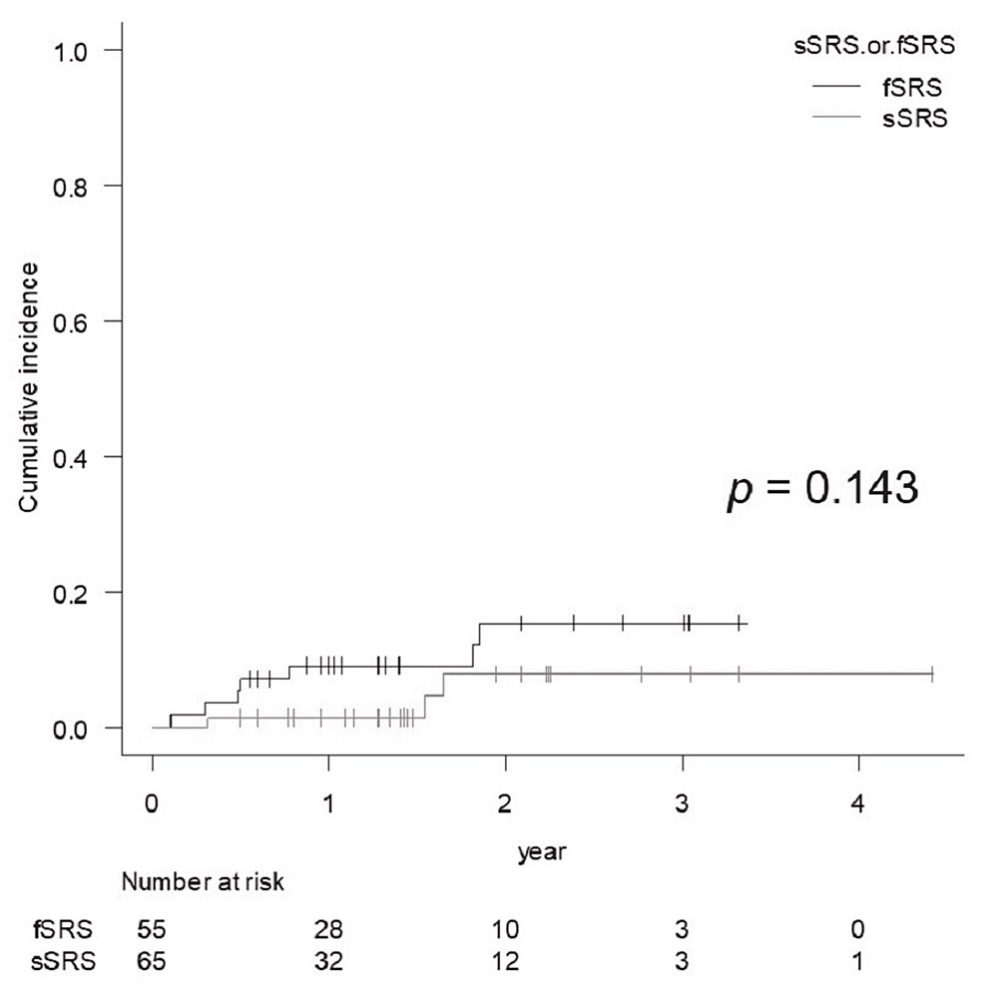
Comparison of the cumulative incidence rate of brain radionecrosis for Grade 2 or more for lesions treated with sSRS and fSRS, for which the diagnoses of BR were based on imaging findings except for 4 cases with surgical resection

Grades 2 and 3 BR occurred in three and seven cases, respectively, of which four cases with four lesions required surgical removal. In one case that operated early (six weeks after STI), pathological diagnosis revealed active tumor cells. In the other three cases, only necrotic tissue was seen, and no viable tumor cells were confirmed. The incidence of BR for Grade ≥2 was not significantly different between the sSRS and fSRS groups (p = 0.143) (Figure 5).

**Figure 6:**
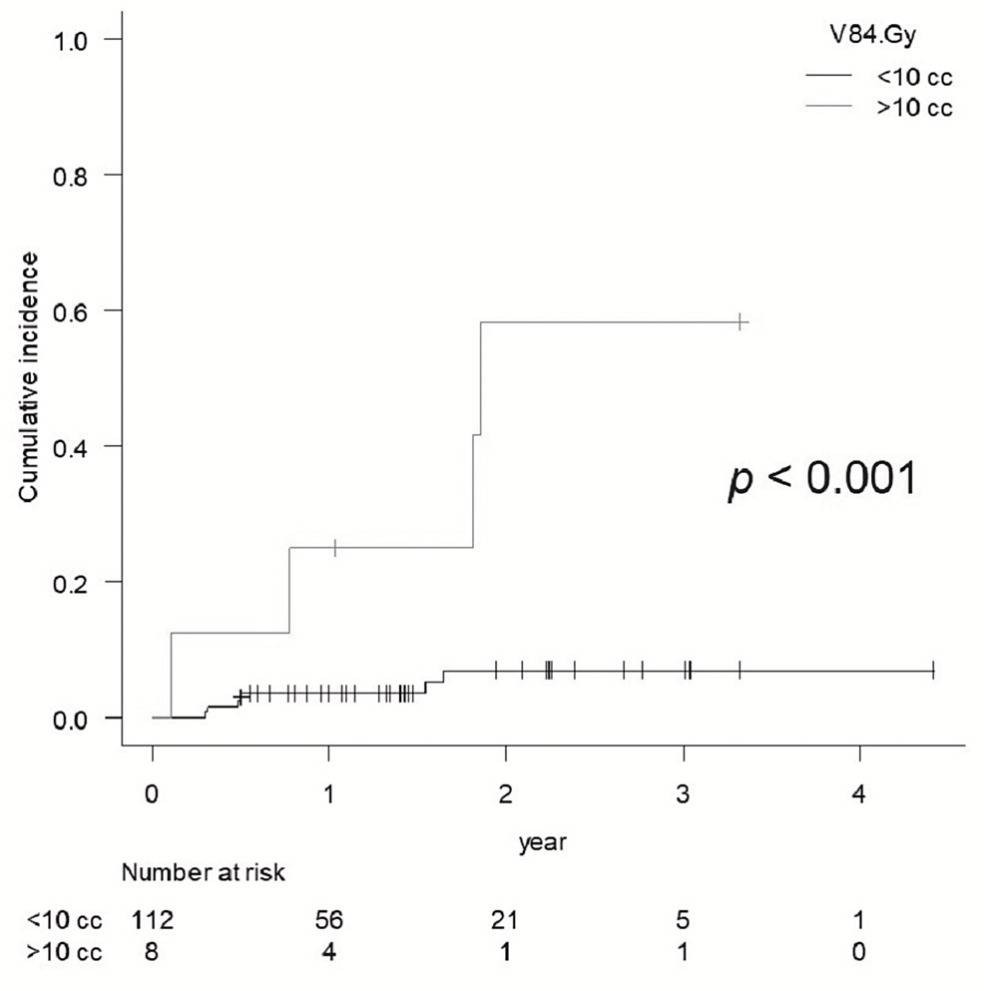
Comparison of the cumulative incidence rate of brain radionecrosis for Grade 2 or more for V84 Gy <10 cc vs. >10 cc

**Figure 7:**
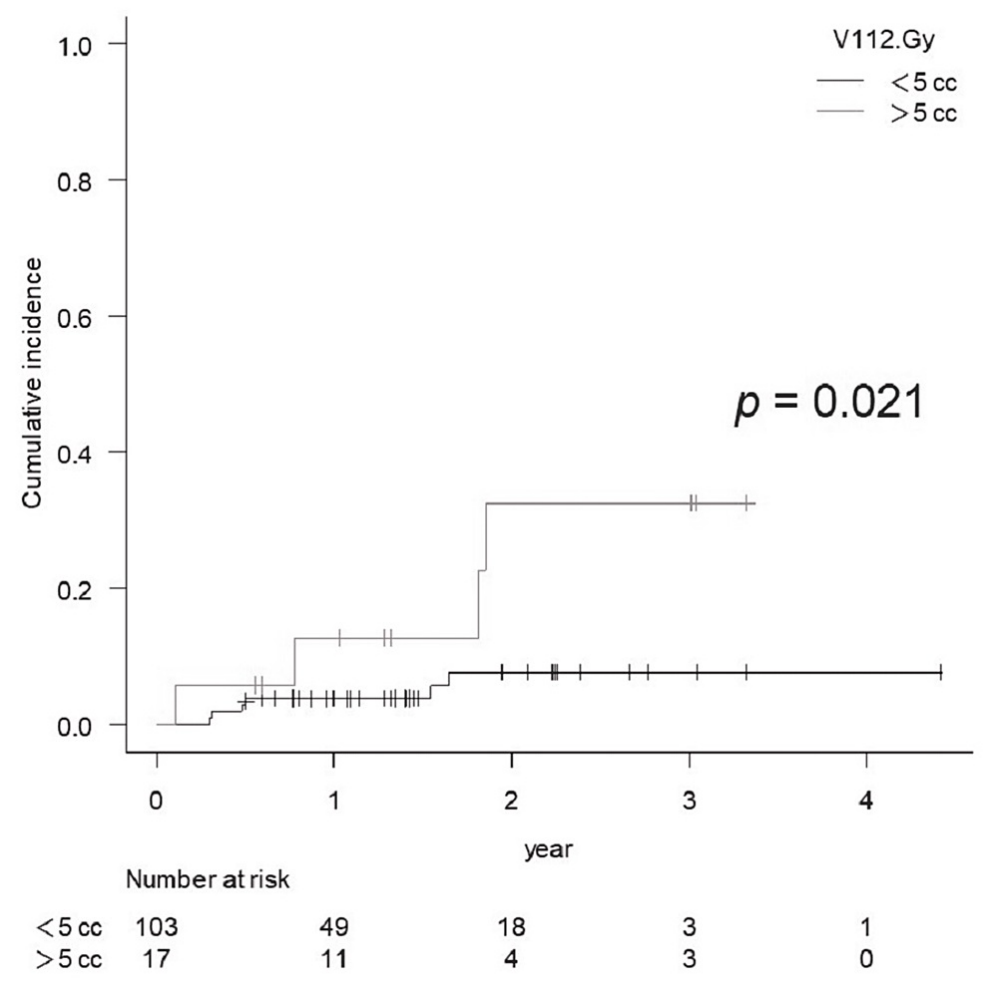
Comparison of the cumulative incidence rate of brain radionecrosis for Grade 2 or more for V112 Gy <5 cc vs. >5 cc

BR of Grade ≥2 was significantly higher for V84 Gy　>10 cc (eight cases, p <0.001) and for V112 Gy >7cc (17 cases, p <0.001), while V112 Gy >5 cc was also statistically significant (p = 0.021) (Figures 6, 7).

BR Grade ≥2 was also significantly higher for GTV >4 cc (p = 0.005).

**Figure 8:**
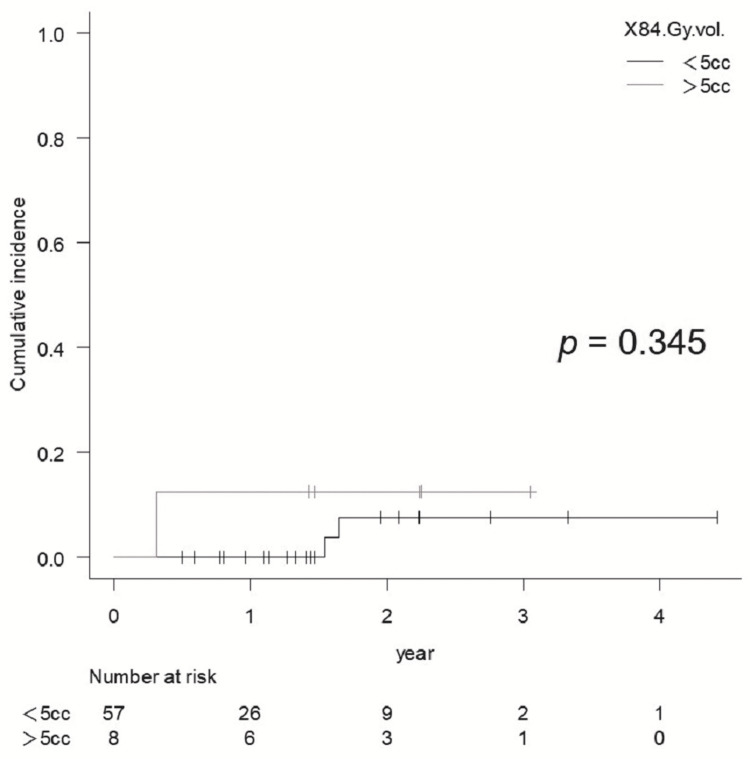
Comparison of the cumulative incidence rate of brain radionecrosis for Grade ≥2 for 84 Gy vol. <5 cc vs. >5 cc in lesions treated with sSRS

**Figure 9:**
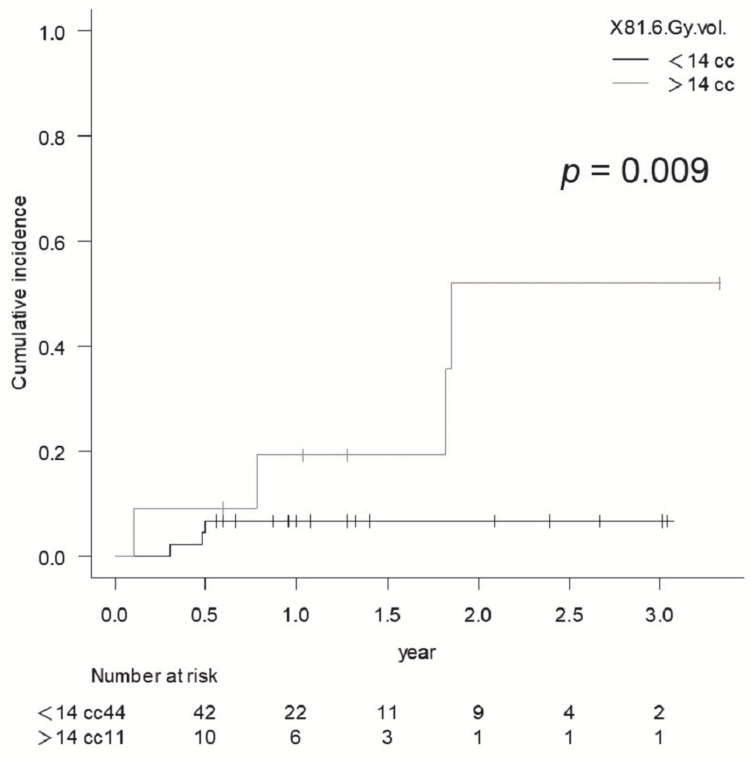
Comparison of the cumulative incidence rate of brain radionecrosis for Grade ≥2 for 81.6 Gy vol. <14 cc vs. >14 cc in lesions treated with fSRS

BED2 84 Gy vol. >5 cc did not show statistical significance for BR of Grade ≥2 in sSRS (p = 0.345) (Figure 8), and BR of Grade ≥2 in fSRS was significantly higher for 81.6 Gy vol. >14 cc (p = 0.009) (Figure 9), while there were no significant differences in the BR of Grade ≥2 with fSRS between 81.6 Gy vol. >20 cc and <20 cc (p = 0.061).

BRRI for fSRS was Grade 3 except for one case with Grade 2. The BR of Grade 3 in the fSRS group was significantly higher for 81.6 Gy vol. >14 cc (p = 0.003), while the BR requiring resection was also significantly higher for 81.6 Gy vol. >14 cc (p = 0.004). The multivariate analysis did not identify any significant factors that contributed to BRRI.

## Discussion

Herein, we report the clinical outcomes based on the initial four-year experience of STI for BMs using Vero4DRT and demonstrated V84 Gy, V112 Gy, and 81.6 Gy vol. as significant indicators of BRRI, along with each threshold volume susceptible to BRRI. Blonigen et al. reported that the incidence of symptomatic BR in sSRS was 10%, and V12 Gy >7.9 cc was a significant threshold, in which 63% had prior WBRT, and any BR occurred in 34.6% and 68.8% for V12 Gy of 4.8-10.8 cc and >10.8 cc, respectively [18]. Minniti et al. reported that the cumulative incidence of BR at one year was 18% in the sSRS group and 9% in the fSRS group [19]. In contrast, the cumulative incidence of symptomatic BR at one year in our series was 5.1% (1.5% for sSRS and 9.3% for fSRS), which was comparable to or rather low. Regarding STI plan optimization, Vero4DRT has several machinery limitations, such as fixed collimator angle and restriction of noncoplanar arc angle (O-ring gantry rotation), and iPlan TPS only provides forward planning of the DCA. Therefore, we have limited sSRS indication and applied fSRS to GTV >1.5 cm to ensure treatment safety. This policy would result in a lower incidence of BRRI, although late asymptomatic radiographical changes, also called radiation effects, are not infrequent for STI due to its ablative nature.

Although several factors have been reported to correlate with BRRI [8], the surrounding brain volumes that received specific doses are the most relevant. Recent studies have suggested that normal brain tissue is much less tolerant of sSRS than the previously established dose-volume tolerance in the RTOG 90-05 trial. Although the National Comprehensive Cancer Network guideline version 1 in 2021 suggested that the indication for fSRS would be GTV >2 cm, we acknowledge that sSRS should be applied to GTV ≤1.5 cm. Therefore, in our series, sSRS was applied to lesions with a GTV maximum diameter of <15 mm and located in non-eloquent areas. The incidence of BRRI was not significantly different between the sSRS and fSRS groups (p = 0.143) (Figure 5), although the fSRS group contained larger lesions. Milano et al. recently reviewed and suggested that a 12 Gy vol. of 5 cc was associated with a risk of symptomatic BR of approximately 10% [11]. In our study, a 12 Gy vol. was ≤7.23 cc (median, 1.76 cc), and the two-year cumulative incidence of symptomatic BR (Grade ≥2) was 7.4% (<10%) for sSRS, which was consistent with the findings of Milano et al. However, no significant differences in the incidence of a BR Grade ≥2 was observed between the 12 Gy vol. (84 Gy vol.) of >5 cc and <5 cc, respectively, probably due to the small number of BRRI cases.

Regarding fSRS, Inoue et al. reported that V23.1 Gy in 3 fr (BED2 112 Gy), V28.8 Gy in 5 fr (BED2 111.7 Gy), and SDE to V14 Gy were correlated with symptomatic BR in the eloquent area, with 7 cc being the significant threshold. The results of this study showed that V112 Gy (SDE to V14 Gy) >7 cc was a significant threshold for BRRI, while V112 Gy (BED2) >5 cc was also statistically significant for BRRI in our series. The threshold volume of V112 Gy might differ depending on the tumor location (e.g., superficial vs. deep).

Milano et al. recently reported that 20 Gy vol. in 3 fr and 24 Gy vol. in 5 fr <20 cc were associated with a <4% risk of BR that required resection [11], while in our series, 81.6 Gy vol. (equivalent to 24 Gy vol. in five fractions) of >14 cc was a significant threshold for BRRI.

Taken together, the dose-volume thresholds relevant for BRRI in our series were rather low compared with those of previous studies. We rigorously evaluated the dose-volume threshold of the surrounding brain by excluding non-parenchymal tissues, which might lead to different results.

One of the main limitations of our study was the potential diagnostic inaccuracy for the pathological condition of enlarging lesions following the nadir response after STI due to the difficulty of differentiating between BR and tumor progression both clinically and radiographically. Similar to previous studies, the number of pathologically verified cases in our series was limited. Telera et al. reported that BR and tumor recurrence were histologically mixed in eight (53%) of 15 patients who underwent surgical excision for enlarging lesions after sSRS [20].

We adopted a GTV D98% to objectively evaluate the near-minimum dose delivered to the GTV margin. The description of near-minimum doses for tumors of extremely small size is still controversial, and the International Commission on Radiation Units and Measurements (ICRU) reports 91 recommended Dv-0.035 cc, instead of D98%, for reporting near-minimum doses in small lesions of <2 cc [21]. Matsuyama and Kogo et al. suggested that PTV (GTV + 1-2 mm) D95% ≥80 Gy (BED10) is associated with a better one-year LC for fSRS (median, 3 fr) for BMs from NSCLC [22]. In their series with favorable LC, the GTV D98% was higher than 80 Gy in most cases. In our study, the one-year LC rate for GTV D98% of >80 Gy was 94.9%, which was comparable to the results of Matsuyama et al., and our results showed that GTV D98% of >80 Gy would lead to better LC. However, the one-year LC rate for all 120 lesions was 87.4%, with GTV D98% (BED10) ranging from 63.9 to 111.7 Gy, and GTV D98% for symptomatic BR was <80 Gy in 70% (Table 3).

**Table 3:** Summary of BR for Grade ≥2 BED: biological effective dose; WBRT: whole-brain radiation therapy.

	Dose regimen	Grade	Resection	Time from STI (week)	Location	GTV diameter (mm)	GTV volume (cc)	GTV D_98 _(Gy, BED10)	V84 Gy (cc)	V112 Gy (cc)	84 Gy vol. (cc) (For sSRS)	81.6 Gy vol. (cc) (For fSRS)	WBRT
1	24 Gy/1 fr	2	no	85	Deep	12	0.15	103.8	1.53	1.15	1.62		no
2	24 Gy/1 fr	2	no	80	Deep	5	0.14	105.1	1.8	1.37	1.95		no
3	24 Gy/1 fr	3	no	16	Superficial	15	1.75	99.9	4.38	3.45	6.12		no
4	35 Gy/ 5 fr	2	no	16	Superficial	5	0.12	76.2	1.68	1.16		1.87	no
5	35 Gy/ 5 fr	3	no	25	Deep	18	1.84	68.1	3.87	2.63		5.83	Before STI
6	35 Gy/ 5 fr	3	no	94	Deep	19	4.97	67.9	12.8	8.42		18.3	no
7	35 Gy/ 5 fr	3	yes	40	Superficial	43	21.51	72.6	21.78	13.58		43.35	no
8	35 Gy/ 5 fr	3	yes	95	Superficial	32	11.54	70.4	14.97	9.29		27.16	no
9	35 Gy/ 5 fr	3	yes	6	Superficial	28	6.18	70.2	10.31	7.19		16.49	no
10	42 Gy/10 fr	3	yes	26	Superficial	16	1.33	72.8	5.99	4.22		7.55	no
Median				25.9		17	1.8	72.7	5.19	3.84	1.95	16.49	

Given the rather low GTV D98% in fSRS (<80 Gy as BED10), we cannot deny the coexistence of a marginal tumor remnant and/or regrowth along with BR in substantial cases with enlarging lesions after nadir response. Furthermore, the accurate discrimination of BR from viable tumor tissue is usually difficult with conventional imaging studies in most radiographically regrowing lesions after maximum response, which is one of our study limitations as previous studies. Although 3,4-dihydroxy-6-(18)F-fluoro-1-phenylalanine (F-DOPA) PET has been reported as a useful tool with high sensitivity and specificity [23,24], we could not use this modality at our institution.

Minniti et al. reported that the one-year LC rate in fSRS (27 Gy in 3 fr) for BM >2cm was 91% [19], which was better than our fSRS group. They used F-DOPA PET for differentiating tumor progression from BR and might more accurately diagnose them than ours. In general, 18 Gy in sSRS for tumors 21 to 30 mm and 27 to 35Gy in 3-5 fraction fSRS for tumors 21 to 40 mm are associated with 75% and 80% one-year LC, respectively [2]. We also defined tumor progression according to the recently proposed more rigorous criteria [17]. The LC rate in our series would therefore seem to be comparable to previous studies. Nonetheless, we should further consider the volume effect of STI to attain a two-year incidence of BRRI <5%, and the dose-fractionation scheme of fSRS to ensure a sufficient GTV dose. More investigations based on long-term follow-up are necessary to elucidate the relevant factors susceptible to BRRI, presumably multifactorial.

## Conclusions

The incidence of BRRI following sSRS and fSRS using Vero4DRT for BMs was comparable to that of the previous LINAC series, and the relevant dose-volume thresholds of the surrounding brain tissue, rather low compared to previous suggestions, were demonstrated. The threshold volume might differ according to tumor location (superficial vs. deep) and/or the observational period. However, this study suggests that the indication for single and ultra-hypofractionated (3-5 fr) SRS should be limited to smaller tumors than previously acknowledged to ensure long-term safety and efficacy. More multifactorial studies based on larger populations and longer follow-up periods are necessary to elucidate the patient and treatment characteristics that are susceptible to BRRI.
